# Supplementary material for: Genome-Wide Analysis of the WOX Transcription Factor Genes in Dendrobium catenatum Lindl
Source: Genes (Basel). 2022 Aug 19;13(8):1481. doi: 10.3390/genes13081481 (PMC9408443; doi:10.3390/genes13081481)
Supplement: Supplementary file 1 [file genes-13-01481-s001.zip › genes-1862141-supplementary.pdf]

Table S1: The accession of *AtWOX* genes.

Gene	Accession
ATWUS	AT2G17950
ATWOX1	AT3G18010
ATWOX2	AT5G59340
ATWOX3	AT2G28610
ATWOX4	AT1G46480
ATWOX5	AT3G11260
ATWOX6	AT2G01500
ATWOX7	AT5G05770
ATWOX8	AT5G45980
ATWOX9	AT2G33880
ATWOX10	AT1G20710
ATWOX11	AT3G03660
ATWOX12	AT5G17810
ATWOX13	AT4G35550
ATWOX14	AT1G20700

Table S2: The primer sequences of *DcaWOXs'* CDS.

Primer Name	Sequence (5′ to 3′)
DcaWOX2-SalI-F	*GCAGCGGCCGTCGAC*ATGGAAGAGGAGAAGC
DcaWOX2-EcoRI-R	*GTTGATTCAGAATTC*TTAGCTCCGGTTATCACC
DcaWOX3a-SalI-F	*GCAGCGGCCGTCGAC*ATGCCTCAAGTTCCATC
DcaWOX3a-EcoRI-R	*GTTGATTCAGAATTC*TTAGTTGGTGGAAGCTGAGC
DcaWOX5-SalI-F	*GCAGCGGCCGTCGAC*TGTGCAACAGCAGAGTG
DcaWOX5-EcoRI-R	*GTTGATTCAGAATTC*TTAGAAGAAGCCCAAGGAGAGG
DcaWOX9-SalI-F	*GCAGCGGCCGTCGAC*ATGGCTTCATCAAACAGGCACTGG
DcaWOX9-EcoRI-R	*GTTGATTCAGAATTC*ATAGGATGCTCCATGCTG
DcaWOX11b-SalI-F	*GCAGCGGCCGTCGAC*GGACAATAACAAACCCCC
DcaWOX11b-EcoRI-R	*GTTGATTCAGAATTC*CTTTCACCCATCTG
DcaWOX13a-SalI-F	*GCAGCGGCCGTCGA*CATGGCATGGGAAAG
DcaWOX13a-EcoRI-R	*GTTGATTCAGAATTC*AACTTCAAATCGCACGCC
